# Growth performance comparison of exclusively breastfed infants with partially breastfed and formula fed infants

**DOI:** 10.1371/journal.pone.0237067

**Published:** 2020-08-20

**Authors:** Xin-Nan Zong, Hui Li, Ya-Qin Zhang, Hua-Hong Wu

**Affiliations:** Department of Growth and Development, Capital Institute of Pediatrics, Beijing, China; University of North Carolina at Greensboro, UNITED STATES

## Abstract

**Background:**

Little consensus exists for growth performance of different feeding patterns in infancy. The objective of this study is to assess the growth performance of exclusively breastfed, partially breastfed and formula fed infants in China.

**Methods:**

Data from a total of 109,052 infants aged 1-<12 months were collected from the 4th and 5th China National Surveys in 2005 and 2015. Feeding patterns were classified into three types for infants under 6 months of age: exclusive breastfeeding, partial breastfeeding and formula feeding. Exclusive breastfeeding refers to feeding exclusively from the mother’s own milk (bottle-feeding included).

**Results:**

34.0% and 43.9% of infants were exclusively breastfed and 41.5% and 36.3% were partially breastfed at 4-<6 months in 2005 and 2015 respectively. Exclusively breastfed infants were generally a little heavier than partially breastfed and formula fed infants aged 1-<6 months; however, there was not a significant statistical difference between continued breastfeeding and formula feeding infants aged 6-<12 months. No significant statistical difference for length was observed among the three groups for ages 1-<6 months; however, infants who were continued to be breastfed were a little shorter compared to those who were formula fed (ages 6-<12 months). For infants aged 1-<2 months there was not a substantial difference from the 2006 WHO growth standards; however, for infants aged 2-<12 months the average weight and length of different feeding infants in China were a little heavier and longer than the 2006 WHO growth standards.

**Conclusions:**

Partial breastfed and formula fed infants were a little lighter than exclusively breastfed infants in the first half of the first year. Formula fed infants were a little longer than continued breastfed infants in the second half.

## Introduction

Breastfeeding not only brings many health benefits to mothers and babies, but also has important social development implications. A meta-analysis indicated protection against child infections and malocclusion, increases in intelligence, and probable reductions in overweight and diabetes [1].

The National Center for Health Statistics (NCHS)/World Health Organization (WHO) growth reference for infants [2,3] is based on the Fels study conducted from 1929 to 1975 and most of the infants in the Fels study were bottle-fed; of those who were breast-fed, very few were breast-fed for more than 3 months [4]. The application of the NCHS/WHO growth reference has led some investigators to conclude that exclusively breast-fed infants begin to falter in growth by the third month after birth [5]. WHO Working Group on Infant Growth further reviewed and inferred that the growth pattern of exclusive breastfeeding infants may be different from the NCHS/WHO growth reference [4]. In the WHO Multicenter Growth Reference Study, three quarters (74.7%) of the infants were exclusively/predominantly breastfed for at least 4 months, 99.5% were started on complementary foods by 6 months of age, and 68.3% were partially breastfed until at least age 12 months [6].

With social development, infant feeding knowledge and childcare skills of the parents have been continuously improving and thus there may be some changes for the growth performance of differently fed infants. A birth cohort in Hong Kong, China suggested that breast feeding may only have short-term effects on physical growth [7]. The Longitudinal Study of Chinese Breastfeeding Infants Growth and Development showed both weight and length did not display substantial differences between exclusively and non-exclusively breastfed infants from birth to 4 months [8].

The benefits of breastfeeding for women and children are well recognized in many respects, but little consensus exists for the growth performance of infants being fed by different modalities. Our aim was to assess the growth performance of exclusive breastfeeding, partial breastfeeding and bottle feeding based on a series of national surveys in China [9,10]. In addition, we also compared average weight and length level of different feeding regimens in China with the 2006 WHO growth standards [11].

## Methods

### Data source

#### Study subject

Data from infants aged 1 to 12 months were obtained from the 4th National Survey on Physical Growth and Development of Children (NSPGDC) in urban and suburban areas of the nine cities of China carried out in 2005 [9] and the 5th NSPGDC in 2015 [10]. Of the nine cities, Beijing and Shanghai are municipalities, and the other seven cities are provincial capital cities including Harbin (Heilongjiang’s provincial capital), Xi'an (Shaanxi), Nanjing (Jiangsu), Wuhan (Hubei), Guangzhou (Guangdong), Fuzhou (Fujian), and Kunming (Yunnan). Urban infants were defined as permanently living in an urban area in the surveyed city, or children who moved into the surveyed city from other large cities and lived in the surveyed city longer than two-thirds of their own ages; and suburban infants were defined as either one or both parents being farmers and where the children were brought up in a suburban rural area (surrounding the surveyed city). Exclusion criteria were: gestational age at birth <37 weeks or birth weight <2,500 g, twins or multiple births, participants suffering from chronic systemic disease, congenital diseases, endocrine diseases, diseases of the nervous system, and those presenting with fever for more than seven days in the past two weeks or continuous diarrhoea more than five times per day for five days or longer. Please refer to the reference [12] for detailed survey methodology. The study protocols of the NSPGDC were approved by the Ethics Committees of the Capital Institute of Pediatrics in Beijing, China. Participation was voluntary and informed consent was sought from all of the respondents.

#### Sampling

The NSPGDC used stratified cluster sampling method according to urban/suburban areas and administrative districts in each of the nine cities. Infants came from the communities (as a minimum cluster unit) in each selected administrative district.

#### Age grouping

Infants aged 1-<12 months were divided into eight groups: monthly for 1-<6 months (1-<2, 2-<3, 3-<4, 4-<5, 5-<6) and bimonthly for 6-<12 months (6-<8, 8-<10, 10-<12). Each sex-age subgroup consisted of almost an equal sample size of 150 to 200 infants in urban and suburban areas of each of the nine cities.

#### Sample size

**Fig 1:**
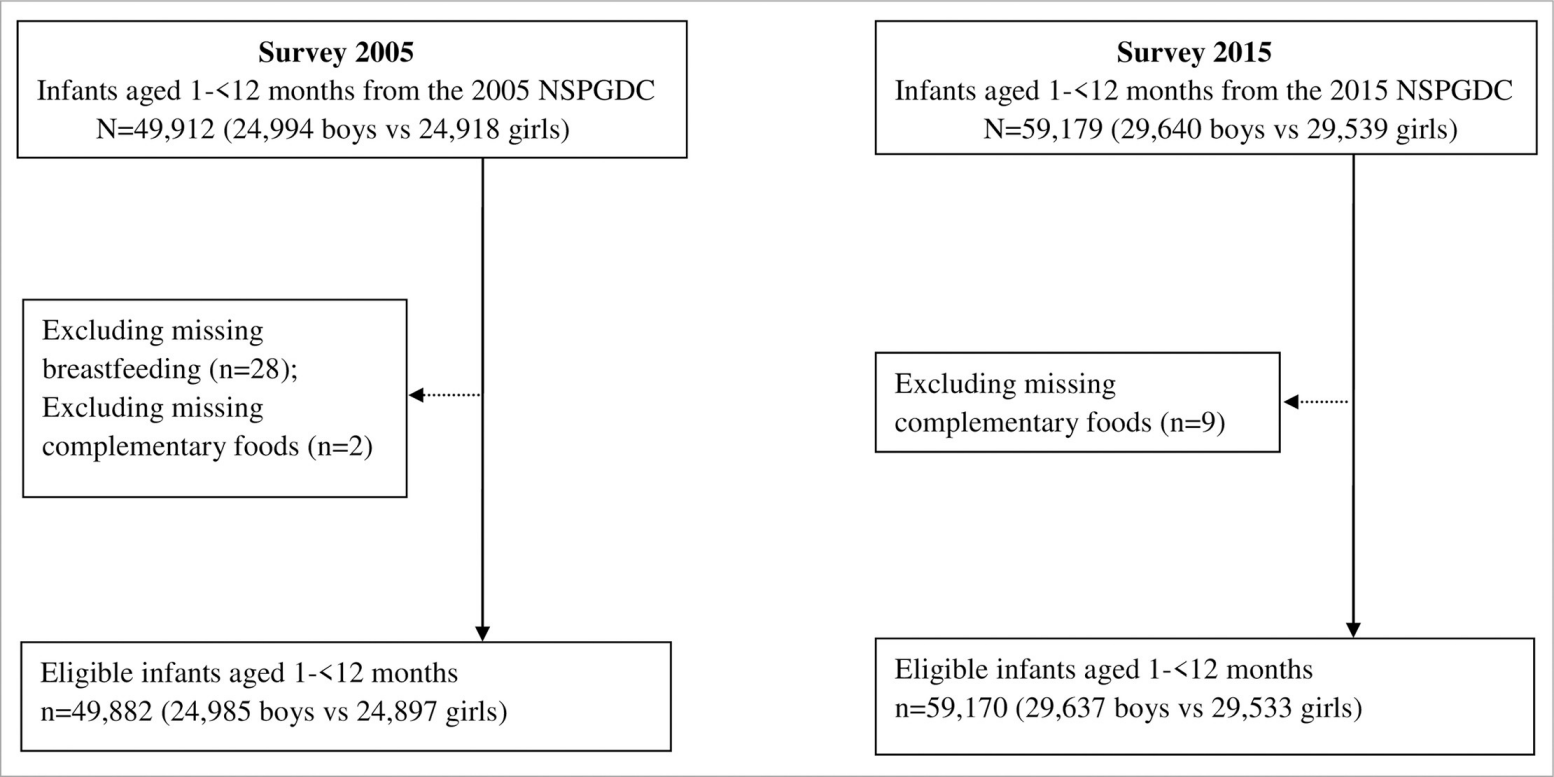
Flow chart of the study population in the 2005 and 2015 surveys.

A total of 49,912 infants with 24,994 boys and 24,918 girls were collected from the 4th NSPGDC in 2005 and a total of 59,179 infants with 29,640 boys and 29,539 girls from the 5th NSPGDC in 2015. After excluding missing breastfeeding and complementary foods, eligible 49,882 infants with 24,985 boys and 24,897 girls from the 4th NSPGDC and eligible 59,170 infants with 29,637 boys and 29,533 girls from the 5th NSPGDC contributed to this study. Fig 1 showed flowchart of the study population in the 2005 and 2015 surveys.

### Anthropometric measurement

Body weight and length were measured using unified measuring tools/instruments in a standardized way by specially trained technicians or nurses. Weight was measured with lever scale to the nearest 0.01 kg with children wearing a diaper, and the lightest vest or shorts. Length was measured with horizontal metal infantometer to the nearest 0.1 cm. Errors of weight and length were not more than 0.05 kg or 0.5 cm between two repeated measurements. All physical measurements were carried out at least one hour after a meal between approximately 8 a.m. and 4 p.m.

### Breastfeeding and complementary foods

Breastfeeding intake investigated breast milk, cows’ milk, formula, cereals (such as rice, porridge and wheat), and water in the previous 24 hours. Breastfeeding data were collected by recall interviews carried out face-to-face with the parents or caregivers of the infants. Questionnaire set one item for every kind of feeding and there were two possible answers: they scored one if child had consumed that item in a specified timescale, and they scored zero if they had not consumed it during that period.

Feeding patterns were classified into three types for infants under 6 months of age: exclusive (or predominant) breastfeeding were those who were exclusively breastfed and sometimes drank plain water, partial breastfeeding were those who received breast milk and formula, and formula feeding were those who only received formula. Exclusive breasting refers to feeding exclusively from the mother’s own milk (inclusive of mother’s own milk by bottle). Infants under 6 months of age who received any complementary foods would be divided into partial breastfeeding group.

### Statistical analysis

Considering secular positive growth trend of weight and length in China [13], data analysis was performed by the division of the 2005 and 2015 NSPGDC. Proportion of exclusive breastfeeding, partial breastfeeding and formula feeding was tested using Chi-square test, with statistical significance set at 0.05. Average weight and length among different feeding infants were compared using t-test or analysis of variance (ANOVA), with statistical significance set at 0.05. Differences between average weight and length of different feeding infants in China and the 2006 WHO growth standards were assessed. Data analysis was performed in SAS v9.4 (SAS Institute, Cary, North Carolina).

## Results

### Characteristic of breastfeeding and complementary foods

34.0% and 43.9% of infants were exclusively breastfed, and 41.5% and 36.3% of infants were partially breastfed at 4-<6 months of age in 2005 and 2015 respectively (Table 1). 39.6% and 47.3% of infants continued to partially breastfeed to 10-<12 months in 2005 and 2015 respectively (Table 2).

**Table 1:** Proportion of breastfeeding in infants aged 1-<6 months in China.

Age (months)	Feeding patterns				
	Sample size	Exclusive breastfeeding	Partial breastfeeding	Formula feeding	p-value
*Survey 2005*					
1-<2	6236	57.5	32.8	9.7	<0.0001
2-<3	6121	55.8	30.2	14.0	<0.0001
3-<4	6213	51.9	31.4	16.7	<0.0001
4-<5	6268	39.1	38.9	22.0	<0.0001
5-<6	6200	28.9	44.2	26.9	<0.0001
1-<4	18570	55.1	31.5	13.4	<0.0001
4-<6	12468	34.0	41.5	24.5	<0.0001
1-<6	31038	46.6	35.5	17.9	<0.0001
*Survey 2015*					
1-<2	7417	58.1	34.9	7.0	<0.0001
2-<3	7267	60.0	29.7	10.3	<0.0001
3-<4	7448	60.6	26.3	13.1	<0.0001
4-<5	7219	51.5	31.1	17.4	<0.0001
5-<6	7240	36.3	41.4	22.3	<0.0001
1-<4	22132	59.6	30.3	10.1	<0.0001
4-<6	14459	43.9	36.3	19.8	<0.0001
1-<6	36591	53.4	32.6	14.0	<0.0001

*χ*^2^ test for comparison of proportion of three feeding types.

**Table 2:** Proportion of continued breastfeeding in infants aged 6-<12 months in China.

Age (months)	Feeding patterns			
	Sample size	Continued breastfeeding	Formula feeding	p-value
*Survey 2005*				
6-<8	6316	63.8	36.2	<0.0001
8-<10	6305	52.8	47.2	<0.0001
10-<12	6223	39.6	60.4	<0.0001
6-<12	18844	52.1	47.9	<0.0001
*Survey 2015*
6-<8	7550	72.0	28.0	<0.0001
8-<10	7531	60.3	39.7	<0.0001
10-<12	7498	47.3	52.7	<0.0001
6-<12	22579	59.9	40.1	<0.0001

*χ*^2^ test for comparison of proportion of three feeding types.

### Comparison of weight and length among different feeding infants

Exclusively breastfed infants were generally a little heavier than partially breastfed infants and the latter were generally a little heavier than formula feeding infants aged 1-<6 months. The differences of average weight between exclusive breastfeeding and formula feeding groups ranged from 0.16 kg to 0.27 kg by age in 2005 and from 0.09 kg to 0.22 kg in 2015 (Table 3). For infants aged 6-<12 months, there was not a significant statistical difference for average weight between continued breastfeeding and formula feeding infants (Table 4).

**Table 3:** Comparison of mean weight (kg) among three feeding types for infants aged 1-<6 months in China.

Age (months)	Exclusive breastfeeding (mean (SD))	Partial breastfeeding (mean (SD))	Formula feeding (mean (SD))	Dif1	Dif2	Dif3	p-value
*Survey 2005*							
*Boys*							
1-<2	5.17(0.70)	5.06(0.66)	5.00(0.71)	0.11	0.17	0.06	<0.0001
2-<3	6.34(0.73)	6.23(0.74)	6.14(0.74)	0.11	0.20	0.09	<0.0001
3-<4	7.20(0.81)	7.08(0.79)	6.97(0.76)	0.12	0.23	0.11	<0.0001
4-<5	7.77(0.88)	7.70(0.88)	7.55(0.87)	0.07	0.22	0.15	<0.0001
5-<6	8.37(0.97)	8.21(0.95)	8.15(0.89)	0.16	0.22	0.06	<0.0001
*Girls*							
1-<2	4.80(0.60)	4.73(0.57)	4.62(0.64)	0.07	0.18	0.11	<0.0001
2-<3	5.80(0.72)	5.70(0.67)	5.64(0.65)	0.10	0.16	0.06	<0.0001
3-<4	6.63(0.76)	6.47(0.72)	6.37(0.71)	0.16	0.26	0.10	<0.0001
4-<5	7.21(0.82)	7.09(0.81)	7.01(0.77)	0.12	0.20	0.08	<0.0001
5-<6	7.75(0.90)	7.56(0.85)	7.48(0.88)	0.19	0.27	0.08	<0.0001
*Survey 2015*							
*Boys*							
1-<2	5.01(0.63)	4.94(0.59)	4.92(0.63)	0.07	0.09	0.02	0.0009
2-<3	6.28(0.74)	6.17(0.73)	6.18(0.70)	0.11	0.10	-0.02	<0.0001
3-<4	7.18(0.82)	7.03(0.81)	7.01(0.77)	0.15	0.17	0.02	<0.0001
4-<5	7.86(0.94)	7.69(0.85)	7.68(0.87)	0.17	0.18	0.01	<0.0001
5-<6	8.32(0.96)	8.21(0.99)	8.16(0.91)	0.12	0.17	0.05	0.0002
*Girls*							
1-<2	4.70(0.58)	4.62(0.59)	4.61(0.58)	0.08	0.09	0.01	<0.0001
2-<3	5.80(0.67)	5.63(0.64)	5.66(0.66)	0.17	0.14	-0.03	<0.0001
3-<4	6.56(0.75)	6.45(0.71)	6.37(0.71)	0.10	0.19	0.09	<0.0001
4-<5	7.21(0.84)	6.99(0.76)	7.01(0.76)	0.22	0.20	-0.02	<0.0001
5-<6	7.71(0.91)	7.54(0.85)	7.49(0.84)	0.17	0.22	0.05	<0.0001

SD, Standard deviation; Dif1 indicates the difference of mean weight of exclusive breastfeeding minus partial breastfeeding infants; Dif2 indicates the difference of mean weight of exclusive breastfeeding minus formula feeding infants; Dif3 indicates the difference of mean weight of partial breastfeeding minus formula feeding infants; ANOVA for comparison of mean weight among three feeding types.

**Table 4:** Comparison of mean weight (kg) and length (cm) between two feeding types for infants aged 6-<12 months in China.

Age (months)	Weight				Length			
	Continued breastfeeding (mean (SD))	Formula feeding (mean (SD))	Dif	p-value	Continued breastfeeding (mean (SD))	Formula feeding (mean (SD))	Dif	p-value
*Survey 2005*								
*Boys*								
6-<8	8.69(1.05)	8.62(0.97)	0.07	0.0536	69.4(2.5)	69.6(2.6)	-0.2	0.0313
8-<10	9.30(1.09)	9.23(1.01)	0.07	0.0709	72.3(2.6)	72.4(2.6)	-0.1	0.1530
10-<12	9.76(1.14)	9.80(1.08)	-0.04	0.3005	74.9(2.8)	75.2(2.7)	-0.3	0.0020
*Girls*								
6-<8	8.09(0.96)	8.00(0.89)	0.09	0.0074	67.8(2.5)	68.0(2.5)	-0.2	0.0207
8-<10	8.66(1.04)	8.62(1.00)	0.04	0.2091	70.6(2.6)	70.9(2.6)	-0.3	0.0020
10-<12	9.16(1.08)	9.12(1.00)	0.04	0.2942	73.4(2.8)	73.6(2.7)	-0.2	0.0516
*Survey 2015*								
*Boys*								
6-<8	8.71(1.00)	8.65(1.00)	0.06	0.0930	69.4(2.4)	69.7(2.5)	-0.3	0.0020
8-<10	9.31(1.05)	9.26(1.05)	0.05	0.1878	72.2(2.5)	72.5(2.5)	-0.3	0.0011
10-<12	9.87(1.13)	9.80(1.09)	0.07	0.0503	74.9(2.6)	75.0(2.6)	-0.1	0.0936
*Girls*								
6-<8	8.08(0.94)	7.98(0.93)	0.10	0.0018	67.8(2.4)	67.9(2.4)	-0.1	0.1258
8-<10	8.68(1.04)	8.63(1.00)	0.05	0.1352	70.7(2.6)	70.9(2.6)	-0.2	0.0257
10-<12	9.18(1.08)	9.16(1.03)	0.02	0.4267	73.4(2.6)	73.6(2.6)	-0.2	0.0182

SD, Standard deviation; Dif indicates the differences of mean weight and length of continued breastfeeding minus formula feeding infants; t-test for comparison of mean weight and length between continued breastfeeding and formula feeding infants.

No significant statistical difference for average length was found among the three categories aged 1-<6 months (Table 5). For infants aged 6-<12 months, continued breastfeeding compared to continued formula feeding resulted in slightly shorter infants, with the differences ranging from -0.3 cm to -0.1 cm during 6-<12 months (Table 4).

**Table 5:** Comparison of mean length (cm) among three feeding types for infants aged 1-<6 months in China.

Age (months)	Exclusive breastfeeding (mean (SD))	Partial breastfeeding (mean (SD))	Formula feeding (mean (SD))	Dif1	Dif2	Dif3	p-value
*Survey 2005*							
*Boys*							
1-<2	56.8(2.4)	56.6(2.4)	56.6(2.5)	0.2	0.2	0.0	0.1058
2-<3	60.6(2.3)	60.5(2.3)	60.4(2.5)	0.1	0.2	0.1	0.3181
3-<4	63.3(2.3)	63.1(2.2)	63.0(2.2)	0.2	0.3	0.1	0.0140
4-<5	65.3(2.3)	65.4(2.3)	65.3(2.3)	-0.1	0.0	0.1	0.5121
5-<6	67.4(2.4)	67.4(2.3)	67.5(2.4)	0.0	-0.1	-0.1	0.4762
*Girls*							
1-<2	55.6(2.2)	55.6(2.1)	55.4(2.5)	0.0	0.2	0.2	0.1296
2-<3	59.1(2.4)	59.0(2.4)	59.0(2.2)	0.1	0.1	0.0	0.2299
3-<4	61.9(2.2)	61.8(2.2)	61.7(2.1)	0.1	0.2	0.1	0.3875
4-<5	63.9(2.2)	63.9(2.3)	64.0(2.3)	0.0	-0.1	-0.1	0.5070
5-<6	65.8(2.4)	65.8(2.3)	65.9(2.4)	0.0	-0.1	-0.1	0.5250
*Survey 2015*							
*Boys*							
1-<2	56.3(2.1)	56.2(2.1)	56.3(2.1)	0.1	0.0	-0.1	0.4206
2-<3	60.4(2.2)	60.2(2.3)	60.4(2.4)	0.2	0.0	-0.2	0.0555
3-<4	63.4(2.3)	63.3(2.2)	63.4(2.1)	0.1	0.0	-0.1	0.4676
4-<5	65.7(2.3)	65.6(2.2)	65.8(2.3)	0.1	-0.1	-0.2	0.2822
5-<6	67.6(2.4)	67.6(2.3)	67.8(2.3)	0.0	-0.1	-0.2	0.1892
*Girls*							
1-<2	55.3(2.1)	55.1(2.1)	55.1(2.1)	0.2	0.2	0.0	0.0369
2-<3	59.1(2.2)	58.6(2.1)	58.9(2.2)	0.5	0.2	-0.2	<0.0001
3-<4	61.8(2.2)	61.8(2.1)	61.8(2.2)	0.1	0.1	0.0	0.6856
4-<5	64.1(2.2)	63.9(2.1)	64.1(2.2)	0.2	0.1	-0.2	0.0119
5-<6	66.0(2.4)	66.0(2.2)	66.0(2.3)	0.0	0.0	0.0	0.9749

SD, Standard deviation; Dif1 indicates the difference of mean length of exclusive breastfeeding minus partial breastfeeding infants; Dif2 indicates the difference of mean length of exclusive breastfeeding minus formula feeding infants; Dif3 indicates the difference of mean length of partial breastfeeding minus formula feeding infants; ANOVA for comparison of mean length among three feeding types.

### Sensitivity analysis of weight and length of different feeding infants with the 2006 WHO growth standards

Comparing with the 2006 WHO growth standards, there was not a substantial difference for average weight and length of different feeding infants aged 1-<2 months; however, for infants from 2-<3 months to 10-<12 months, the average weight and length of different feeding infants in China were a little heavier and longer than the 2006 WHO growth standards (Tables 6 and 7).

**Table 6:** Mean differences of weight (kg) and length (cm) of three feeding types for infants aged 1-<6 months between this study and the 2006 WHO growth standards ($\bar{x}(95\%CI)$).

Age (months)	Boys			Girls		
	Exclusive breastfeeding	Partial breastfeeding	Formula feeding	Exclusive breastfeeding	Partial breastfeeding	Formula feeding
*Survey 2005*						
*Weight*						
1-<2	0.15(0.12,0.18)	0.04(0.01,0.08)	-0.02(-0.10,0.07)	0.14(0.11,0.17)	0.07(0.03,0.11)	-0.04(-0.11,0.03)
2-<3	0.37(0.34,0.41)	0.26(0.21,0.30)	0.17(0.09,0.24)	0.31(0.28,0.35)	0.22(0.17,0.26)	0.16(0.09,0.22)
3-<4	0.51(0.47,0.55)	0.39(0.34,0.44)	0.28(0.22,0.35)	0.49(0.45,0.53)	0.33(0.28,0.38)	0.24(0.18,0.30)
4-<5	0.52(0.47,0.57)	0.44(0.40,0.49)	0.29(0.23,0.35)	0.54(0.50,0.59)	0.43(0.39,0.48)	0.35(0.29,0.41)
5-<6	0.65(0.58,0.71)	0.49(0.44,0.54)	0.43(0.37,0.48)	0.66(0.60,0.72)	0.46(0.42,0.51)	0.38(0.32,0.44)
*Length*						
1-<2	0.2(0.1,0.3)	0.0(-0.1,0.2)	0.0(-0.3,0.3)	0.3(0.2,0.4)	0.2(0.0,0.3)	0.0(-0.3,0.3)
2-<3	0.6(0.5,0.7)	0.6(0.4,0.7)	0.4(0.2,0.7)	0.7(0.6,0.8)	0.5(0.4,0.7)	0.6(0.4,0.8)
3-<4	0.6(0.5,0.8)	0.4(0.3,0.6)	0.4(0.2,0.6)	0.9(0.8,1.0)	0.9(0.7,1.0)	0.8(0.6,1.0)
4-<5	0.4(0.3,0.6)	0.5(0.4,0.7)	0.4(0.3,0.6)	0.9(0.7,1.0)	0.8(0.7,0.9)	0.9(0.7,1.1)
5-<6	0.6(0.4,0.8)	0.6(0.5,0.7)	0.7(0.6,0.9)	0.9(0.8,1.1)	0.9(0.8,1.0)	1.0(0.9,1.2)
*Survey 2015*						
*Weight*						
1-<2	0.00(-0.03,0.02)	-0.08(-0.11,-0.04)	-0.10(-0.17,-0.02)	0.04(0.02,0.07)	-0.04(-0.07,-0.01)	-0.05(-0.13,0.02)
2-<3	0.31(0.28,0.34)	0.20(0.16,0.24)	0.21(0.14,0.28)	0.31(0.28,0.34)	0.14(0.10,0.18)	0.17(0.10,0.24)
3-<4	0.49(0.46,0.53)	0.34(0.30,0.39)	0.32(0.26,0.39)	0.42(0.39,0.45)	0.32(0.27,0.37)	0.23(0.17,0.30)
4-<5	0.60(0.56,0.64)	0.43(0.38,0.48)	0.42(0.35,0.49)	0.54(0.51,0.58)	0.33(0.28,0.37)	0.34(0.28,0.41)
5-<6	0.60(0.55,0.66)	0.49(0.44,0.53)	0.44(0.37,0.50)	0.61(0.57,0.66)	0.44(0.40,0.49)	0.39(0.33,0.45)
*Length*						
1-<2	-0.3(-0.4,-0.2)	-0.4(-0.5,-0.2)	-0.3(-0.6,-0.1)	-0.1(-0.1,0.0)	-0.2(-0.4,-0.1)	-0.3(-0.5,0.0)
2-<3	0.5(0.4,0.6)	0.3(0.1,0.4)	0.5(0.2,0.7)	0.7(0.6,0.8)	0.2(0.1,0.3)	0.4(0.2,0.7)
3-<4	0.7(0.6,0.8)	0.6(0.5,0.8)	0.8(0.6,0.9)	0.9(0.8,1.0)	0.8(0.7,1.0)	0.8(0.6,1.0)
4-<5	0.8(0.7,0.9)	0.7(0.6,0.9)	0.9(0.7,1.1)	1.1(1.0,1.2)	0.8(0.7,0.9)	1.0(0.8,1.2)
5-<6	0.9(0.7,1.0)	0.8(0.7,0.9)	1.0(0.9,1.2)	1.1(1.0,1.2)	1.1(1.0,1.2)	1.1(1.0,1.3)

CI, confidence interval.

**Table 7:** Mean differences of weight (kg) and length (cm) of two feeding types for infants aged 6-<12 months between this study and the 2006 WHO growth standards ($\bar{x}(95\%CI)$).

Age (months)	Boys		Girls	
	Continued breastfeeding	Formula feeding	Continued breastfeeding	Formula feeding
*Survey 2005*				
*Weight*				
6-<8	0.39(0.35,0.44)	0.32(0.26,0.38)	0.44(0.40,0.49)	0.35(0.30,0.41)
8-<10	0.40(0.34,0.45)	0.33(0.28,0.38)	0.44(0.39,0.49)	0.39(0.34,0.44)
10-<12	0.35(0.29,0.41)	0.39(0.34,0.44)	0.44(0.38,0.51)	0.40(0.36,0.45)
*Length*				
6-<8	0.2(0.1,0.4)	0.4(0.3,0.6)	0.5(0.4,0.6)	0.7(0.6,0.9)
8-<10	0.3(0.2,0.4)	0.5(0.3,0.6)	0.5(0.4,0.6)	0.8(0.6,0.9)
10-<12	0.4(0.2,0.5)	0.7(0.6,0.8)	0.6(0.5,0.8)	0.8(0.7,0.9)
*Survey 2015*				
*Weight*				
6-<8	0.41(0.37,0.45)	0.35(0.29,0.41)	0.44(0.40,0.48)	0.33(0.28,0.39)
8-<10	0.41(0.37,0.45)	0.36(0.31,0.42)	0.45(0.41,0.50)	0.40(0.35,0.45)
10-<12	0.46(0.41,0.51)	0.39(0.34,0.44)	0.47(0.42,0.52)	0.44(0.39,0.48)
*Length*				
6-<8	0.2(0.2,0.3)	0.5(0.4,0.7)	0.5(0.4,0.6)	0.7(0.5,0.8)
8-<10	0.3(0.2,0.4)	0.5(0.4,0.7)	0.6(0.5,0.7)	0.8(0.6,0.9)
10-<12	0.4(0.2,0.5)	0.5(0.4,0.6)	0.6(0.5,0.7)	0.8(0.7,0.9)

CI, confidence interval.

## Discussion

Our study showed that the average proportion of both exclusive and partial breastfeeding were 82.1% for infants within 1-<6 months in 2005 and 86.0% in 2015, proving breastfeeding is the main feeding pattern in Chinese infants, which was consistent with other surveys in China [14,15]. Our comparative analysis showed that breastfeeding in China in 2015 was similar to those in developed countries [16]. During 2005–2015 the average proportion of exclusive breastfeeding increased from 55.1% to 59.6% within 1-<4 months and from 34.0% to 43.9% within 4-<6 months, suggesting the implementation of Global Strategy for Infant and Young Child Feeding issued in 2002 [17] and China Feeding Strategy for Infant and Young Child in 2007 [18] have generated a positive impact on improving breastfeeding, especially exclusive breastfeeding.

According to the 4th and 5th NSPGDC, we found the growth performance of formula fed infants fell behind partially breastfed infants and further partial breastfeeding fell behind exclusive breastfeeding for infants aged 1-<6 months. Our finding suggested that breastfeeding, especially exclusive breastfeeding, plays an important role in promoting early growth and development of infants in the first half of the first year.

The 2006 WHO growth standards are considered to represent the growth level of a well-nourished population and the proportion of exclusive breastfeeding in the reference sample is 74.7% within 4 months and the proportion of continued breastfeeding 68.3% to 12 months of age [6]. In China the sample population of the NSPGDC represented a relatively well-nourished population and the percentile reference values from the NSPGDC were used as reference data for assessing Chinese children [19,20]. Both the 4th and 5th NSPGDC verified three different feeding types of infants in China were a little heavier and longer than the 2006 WHO growth standards and were similar to the growth patterns of the 2006 WHO growth standards, indicating different feeding infants can reach the growth level of a well-nourished population, further suggesting partial breastfeeding and formula feeding can also help infants basically thrive and achieve their growth potential. However, bottle feeding during the first year of life may be associated with infant rapid weight gain [21].

WHO Multicentre Growth Reference Study Group hypothesizes that children in all countries can achieve their full growth potential when their nurturing follows health recommendations and care practices [22]. In the reference sample of the 2006 WHO growth standards, the maximum differences of average length among six countries were 1.4 cm, 1.8 cm and 1.0 cm at birth, 6-month and 12-month respectively [23]. Both the 4th and 5th NSPGDC showed that the growth performance of different feeding infants was similar to each other. The maximum differences of average length were not more than 0.2 cm among three feeding types at 1-<6 months and not more than 0.3 cm between bottle fed and continuously breastfed infants at 6-<12 months, suggesting that the variation of growth performance attributed to feeding patterns was less than that to races and regions in infancy.

Our study used two recent large-scale cross-sectional national data to repeatedly validate a small discrepancy of physical growth of differently fed infants in China, which would further enhance the reliability of the results and conclusion. Our limitation was classifying feeding patterns based on breastfeeding intake status without considering complementary foods introduction. The 24 hour recall period may overestimate breastfeeding prevalence because infants may be given supplements at some earlier time [24]. We only examined the growth performance of different feeding types in infancy, and the long-term effects of different feeding types may need to be further studied.

Based on repeated national cross-sectional surveys in China, the growth performance of partially breastfed and formula fed infants slightly fell behind exclusively breastfed infants in the first half of the first year, and the growth performance of formula fed infants was no less favorable than continuously breastfed infants in the second half. More knowledge was added to fully understand and objectively assess the growth performance of different feeding infants in the first and second halves of the first year.
